# Tracking dengue virus type 1 genetic diversity during lineage replacement in an hyperendemic area in Colombia

**DOI:** 10.1371/journal.pone.0212947

**Published:** 2019-03-07

**Authors:** Mauricio A. Salvo, Matthew T. Aliota, Louise H. Moncla, Ivan D. Velez, Andrea I. Trujillo, Thomas C. Friedrich, Jorge E. Osorio

**Affiliations:** 1 Department of Pathobiological Sciences, University of Wisconsin-Madison, Madison, Wisconsin, United States of America; 2 Programa de Estudio y Control de Enfermedades Tropicales (PECET), Universidad de Antioquia, Medellin, Colombia; 3 Wisconsin National Primate Research Center, University of Wisconsin-Madison, Madison, Wisconsin, United States of America; Johns Hopkins University, Bloomberg School of Public Health, UNITED STATES

## Abstract

Dengue virus (DENV) is a flavivirus responsible for the most common and burdensome arthropod-borne viral disease of humans[1]. DENV evolution has been extensively studied on broad geographic and time scales, using sequences from a single gene[2,3]. It is believed that DENV evolution in humans is dominated primarily by purifying selection due to the constraint of maintaining fitness in both humans and mosquitoes[4,5]. Few studies have explored DENV evolutionary dynamics using whole genome sequences, nor have they explored changes in viral diversity that occur during intra-epidemic periods. We used deep sequencing of the viral coding region to characterize DENV-1 evolution in a Colombian population sampled during two high-prevalence dengue seasons in which serotype dominance shifted. Our data demonstrate patterns of strain extinction and replacement within DENV-1 as its prevalence waned and DENV-3 became established. A comparison of whole-genome versus single-gene-based phylogenetic analyses highlights an important difference in evolutionary patterns. We report a trend of higher nonsynonymous to synonymous diversity ratios among non-structural (NS) genes, and statistically significantly higher values among these ratios in the NS1 gene after DENV-1 strain replacement. These results suggest that positive selection could be driving DENV evolution within individual communities. Signals of positive selection coming from distinct samples may be drowned out when combining multiple regions with differing patterns of endemic transmission as commonly done by large-scale geo-temporal assessments. Here, we frame our findings within a small, local transmission history which aids significance. Moreover, these data suggest that the NS1 gene, rather than the E gene, may be a target of positive selection, although not mutually exclusive, and potentially useful sentinel of adaptive changes at the population level.

## Introduction

Dengue virus (DENV; *Flaviviridae; Flavivirus*) is the cause of the most common and most important arthropod-borne viral disease of humans. The DENV complex is composed of four serotypes (DENV-1 to DENV-4) which cause the same clinical manifestations and show similar patterns of systemic dissemination, with tropism principally for monocytes, macrophages, and dendritic cells[1]. DENVs are predominantly transmitted by the mosquitoes *Aedes aegypti* and *Aedes albopictus*, facilitating heavy viral transmission in densely populated tropical and subtropical regions[6,7]. Furthermore, *Ae*. *aegypti* populations have reestablished themselves in the Americas since the cessation of eradication programs in the 1970s, and currently these mosquitoes can be found from the southern United States to north of the Southern Cone [1,8]. This expanding range increasingly potentiates DENV transmission in the United States. Although most DENV infections are asymptomatic, infection with any one of the four serotypes can result in a spectrum of clinical illness including a self-limited febrile illness termed dengue fever (DF). In a minority of cases it can manifest as a life-threatening vascular leakage syndrome, dengue hemorrhagic fever (DHF), and the often-fatal dengue shock syndrome (DSS). Severe disease is believed to be triggered by cross-reactive antibodies elicited by previous infection with a heterologous serotype[1,9]. For example, primary infection with any one of the four DENV serotypes provides a short initial period of cross-protection among all serotypes and later protective immunity against sequential reinfection with the same serotype [10]. In contrast, reinfection with a different DENV serotype can lead to unusually severe and potentially fatal disease[11,12]. This is particularly worrisome because co-circulation of multiple DENV serotypes has been increasingly reported within endemic areas[8], raising concern that more patients could develop severe disease.

DENV is an RNA virus whose inherent genome replication errors result in extensive genetic diversity within each of the four DENV serotypes, both at the between- and within-host levels[13,14]. The four DENV serotypes are roughly 70% identical at the amino acid level[15,16]. Serotype diversity is often partitioned into phylogenetically discrete clusters of sequences termed genotypes, groups of DENV strains with up to 6% sequence divergence at the nucleotide level[15,16]. Each serotype is composed of multiple genotypes, which likely represent the outcomes of independent evolution following geographic isolation[16–18]. In a given geographic area, individual viral variants appear, circulate for a period of time, and then die out, usually to be replaced by another set of variants. This phenomenon has been termed ‘clade replacement’, and is occasionally linked to changes in virulence[17–20]. Replacement can also occur at the serotype level, with such events being reported for all four globally circulating serotypes every 3–5 years on average [8,21,22]. These shifts in dominance within a human population are in large part thought to be the result of differential susceptibility to cross-reactive immunity from individual serotypes [23]. Despite the link between heterotypic secondary or subsequent infection and severe disease, the viral contributions to natural fluctuations in serotype dominance remain poorly understood.

Both clade replacement and serotype shifts can occur concurrently. For example, in Bangkok, Thailand clade replacement was documented within genotype I of DENV-1 and this was coincident with a decline in the overall prevalence of DENV-1 and the rise of DENV-4 in the population[19]. Although stochastic processes likely play a role in determining the phylogenetic structure of DENV populations, there is growing evidence that natural selection also plays a major role in determining the dynamics of lineage turnover[24,25]. For example, Quiner et al. recently demonstrated that a newly emergent DENV-2 clade in Nicaragua was transmitted earlier in infection and more efficiently by *Ae*. *aegypti* [25] suggesting a difference in fitness between the two clades that resulted in greater transmissibility. Therefore, it has become increasingly important to understand the interplay between epidemiological patterns and viral genetics to improve the development of more effective, locally-adapted prevention and control programs.

Accordingly, we have been monitoring DENV epidemiology in the endemic area of Medellin, Colombia for the past 20 years. Nationwide surveillance data suggest patterns of cyclical shifts in serotype dominance observed every few years (Rita Almanza, personal communication)[26,27]. We have leveraged these surveillance efforts to characterize viral evolution coincident with a change in serotype dominance from DENV-1 (2014) to DENV-3 (2016) in a study-site located in the Paris neighborhood of Bello, a northwest suburb of Medellin, Colombia. As previously described, DENV strain emergence and replacement within all serotypes has been well documented[21,24,25,28,29], yet an understanding of the evolutionary mechanisms by which DENV variants become fixed in a population is unclear. We analyzed individual DENV-1 positive human serum samples via deep sequencing to provide a precise characterization of viral diversity throughout seasons of overall serotype dominance and waning prevalence. By analyzing DENV-1 samples collected over two dengue seasons from a population housed within a two kilometer radius, we identified strain turnover within DENV-1 coinciding with waning serotype prevalence. Analyses of the curated data of complete genome sequences conclude that an increase in nonsynonymous viral genetic diversity, indicative of diversifying positive selection, was associated with the clade replacement and that NS1 may be under strong selection.

## Materials and methods

### Sample and participant characteristics

Samples involved in this study were chosen from among dengue-suspected human serum specimens collected as part of standard diagnostic activities of the Eliminate Dengue Program in Colombia (IRB #24020 approved by Comité de Bioética de Investigación en Humanos de la Sede de Investigación Universitaria CBE-SIU, Universidad de Antioquia 2016). Of the 990 samples received throughout this study period, 34 samples were used for whole-genome deep sequencing and assigned new IDs as indicated in Table 1. Sera positive for DENV-1 via qRT-PCR were selected amongst 1) Those sampled between June of 2014 and July of 2016, and 2) Those having a high viral load (10^4^ to 10^8^ copies/ml). The age and sex distribution among these samples was random. The donor samples were rendered completely anonymous and renumbered prior to preparation of extracted RNA for sequencing with only the week of sampling and DENV infection status retained.

**Table 1:** Barrio Paris, Colombia. DENV-1 positive human serum sample details.

Sample ID	Date Collected	Titer	Average Coverage	Gender	Age
**21**	6/20/2014	7.34x10^5^	3067	Male	26
**48**	7/28/2014	3.36x10^5^	825	Female	35
**53**	8/5/2014	4.07x10^7^	1479	Male	17
**73**	9/1/2014	4.07x10^7^	12307	Male	17
**60**	8/21/2014	4.95x10^6^	29006	Male	15
**70**	8/28/2014	5.67x10^7^	17365	Female	41
**90**	9/12/2014	6.00x10^7^	15795	Female	6
**100**	9/26/2014	1.67x10^8^	9234	Male	8
**104**	9/29/2014	10.00x10^5^	1844	Female	35
**111**	10/1/2014	10.00x10^8^	28732	Female	70
**117**	10/3/2014	10.00x10^5^	2827	Male	7
**121**	10/9/2014	-	7667	Female	28
**127**	10/14/2014	10.00x10^4^	1332	Female	41
**137**	10/20/2014	6.68x10^6^	7807	Male	7
**140**	10/22/2014	2.29x10^6^	102	Female	74
**142**	10/28/2014	1.30x10^4^	415	Female	11
**143**	10/28/2014	2.03x10^5^	3288	Female	5
**154**	11/24/2014	1.37x10^7^	18202	Female	8
**289**	2/1/2016	1.42x10^7^	77	Male	7
**294**	2/8/2016	7.06x10^6^	671	Male	17
**297**	2/9/2016	4.9x10^7^	661	Male	11
**303**	2/15/2016	1.65x10^7^	374	Male	15
**310**	3/4/2016	1.79x10^7^	534	Female	20
**318**	10/03/16	2.15x10^6^	39	Female	11
**326**	3/30/2016	3.56x10^6^	1167	Male	38
**330**	3/30/2016	3.56x10^5^	55	Female	11
**347**	4/19/2016	4.95x10^5^	1910	Female	9
**364**	5/16/2016	2.70x10^6^	219	Male	34
**494**	6/21/2016	9.18x10^6^	1189	Male	33
**521**	6/28/2016	5.04x10^5^	150	Female	53
**528**	6/28/2016	1.05x10^7^	2221	Female	32
**535**	6/29/2016	3.53x10^7^	10154	Male	30
**553**	7/5/2016	7.73x10^7^	5954	Male	30
**589**	7/12/2016	3.88x10^6^	168	Female	30
**634**	7/19/2016	1.73x10^7^	2805	Female	36

### Viral load measurements (qRT-PCR)

A TaqMan quantitative reverse transcriptase reaction (qRT-PCR) based on the CDC’s single reaction multiplex rRT-PCR for ZIKV, chikungunya virus (CHIKV), and DENV (referred to as ZDC assay) [30] was performed on serum samples suspected of DENV infection. All viral load measurements were done in a BSL-2 facility at the University of Antioquia, PECET laboratories in Medellin, Colombia on a Roche LightCycler480 instrument.

First, probe fluorophores and quenchers were redesigned as follows [i.e., Virus name (5’ fluorophore, 3’ quencher)]: DENV-1 (HEX, IBFQ), DENV-2 (TEX615, IBFQ), DENV-3 (Cy3, IBRQ), DENV-4 (TYE665, IBRQ), ZIKV (FAM, IBFQ), CHIKV (Cy5, IBRQ). Primers used for qRT-PCR reaction were identical to those in ZDC assay. For each of the following viruses [i.e., Virus [Strain]]: DENV-1 [16007], DENV-2 [16681], DENV-3 [16562], DENV-4 [1036], CHIKV [99659], ZIKV [H/PF/2013]. PCR amplicons including the T7 promoter sequence, flanking the primer binding sites were generated using Q5 High-Fidelity DNA Polymerase (New England BioLabs, USA). Product was isolated using the MinElute Gel Extraction Kit (Qiagen, USA). *In vitro* transcription was performed using the MEGAscript T7 Transcription kit (Invitrogen, USA) and transcripts were quantified using the NanoDrop 2000 (Thermo Fisher Scientific, USA). A dilution of 1x10^10^ transcript copies/l was made and ten-fold dilutions of this transcript were used as a standard curve. All probes and primers were synthesized by Integrated DNA Technologies (IDT, USA).

Viral RNA was extracted from aliquots of ~50-100l of serum using the QIAmp MinElute virus spin kit (Qiagen). RT-PCR was performed using the TaqMan Fast Virus 1-Step Master Mix under the following thermal conditions: 1 cycle of 50C for 15 minutes and then 95C for 2 minutes followed by 45 cycles of 95C for 15 seconds then 55C for 20 seconds then 68C for 20 seconds. Each reaction contained all primers detailed in ZDC assay as well as the previously described probes at concentrations within the instrument’s guidelines. The standard curve was linear up to 8 orders of magnitude and was sensitive down to 100 copies of RNA transcript per reaction.

### Deep sequencing and sample preparation

All samples were processed through the cDNA generation step in a positively pressurized, anteroom-accessed biosafety level 2 laboratory separated from the main work area. Library preparation and sequencing were all performed in the adjacent biosafety 2 laboratory main area. For each sample, ~500l of sera were centrifuged at 5,000xg at 4C for 5 min to remove residual host cells. The supernatant then was centrifuged for an additional 90 min at 4C at 14,000 rpm in order to concentrate virions. Viral RNA was extracted from human sera using the QIAmp MinElute virus spin kit (Qiagen, Germantown, MD), omitting carrier RNA and the AW1 wash step. Eluted RNA was treated with DNase I (DNA-free, Ambion, USA), and cDNA was synthesized using the double-stranded cDNA synthesis kit (Invitrogen, Grand Island, NY, USA) primed by random hexamers. DNA was purified and concentrated using the Agencourt Ampure XP system (Beckman Coulter, USA). Library preparation was performed using the Nextera XT Sample Preparation Kit (Illumina, San Diego, CA). The barcoded library was subsequently cleaned using the Agencourt Ampure XP system and the product was quantified with the Qubit dsDNA HS Assay Kit (Invitrogen). Determination of average fragment length was performed with the High Sensitivity DNA kit and the 2100 Bioanalyzer (Agilent, USA). Equimolar concentrations of each sample were pooled to a total concentration of 2nM. Pooled samples were run on the Illumina MiSeq using a 600-cycle kit as a 12pM library with a 1% PhiX control. Fastq format was chosen as the output file type.

### Sequence data analysis

All fastq files were processed using CLC Genomics Workbench version 9 (CLC Bio, Denmark). A quality score of Q30 and a minimum length of 100 bases was used to trim reads. Trimmed reads from each sample were then initially mapped to reference sequence GQ868570 in order build an initial consensus sequence, requiring that at least 50% of each read was 80% identical to the reference. In order to assess the composition of abundant RNA in each sample, all trimmed reads were also de-novo assembled and resulting contiguous reads with over 100 average base pair coverage were used as input for a BLAST search. This allowed us to observe the major targets of the unbiased cDNA generation. We did not find any laboratory virus contaminants among our samples. Consensus sequences derived from initial mapping, based on majority base call at every position, were extracted and used as references for a secondary mapping of all trimmed reads. This secondary mapping was used as input for assessment of nucleotide changes. This dataset containing 34 samples has an average sequence depth of 5,418 ± 1,320 for DENV-1 coding genomes. Variant calling was performed on samples with a minimum average coverage of 100 reads per position. Only variants present at a frequency ≥1%, covered by a minimum of 100 reads, with a central base quality score of ≥Q30 were considered for further analysis.

### Sequence data availability

The consensus sequences of the 34 whole-genome samples were deposited in GenBank, with the accession numbers (PENDING).

### Diversity calculation

Diversity calculations were performed on samples with a minimum coverage of 100 reads per site across the translated region. Reference-based mappings were exported from CLC Genomics Workbench as sequence alignment/map (SAM) files and converted to sorted pileup files using SAMtools [31]. Nucleotide diversity (π), measured as the average number of pairwise differences between a set of DNA sequences without regard to a reference sequence, was calculated using PoPoolation 1.2.2 (Institute of Population Genetics, University of Veterinary Medicine, Vienna) [32]. To avoid bias stemming from differences in genome coverage across samples, we subsampled each sample’s assembly to 500x coverage using the subsample-pileup.pl script. The Variance-at-position.pl script was used to calculate π for each gene of each sample, and the Syn-nonsyn-at-position.pl script was used to calculate πN (non-synonymous nucleotide variation) and πS (synonymous nucleotide variation) for each gene of each sample. πN/πS ratios were also calculated for each gene of each sample using the previously calculated πN and πS values. To assess differences in selection pressures between clades, πN/πS values for each gene of each sample were grouped into the separate clades for analysis.

A sliding window analysis, using a window size of 9 codons and a step size of 1 codon, was performed using the Syn-nonsyn-sliding.pl script. Plots rendering πN or πS sliding windows analysis show the value corresponding to the nucleotide site in the middle of the codon of the window. The corrections option was disabled for all π analysis. Data were analyzed using R version 3.2.3 (R foundation for statistical computing).

### Bayesian phylogenetic analysis

For each sample, envelope gene (E) sequences were identified, extracted from full-genome assemblies and aligned using CLC Genomics Workbench. A data set of DENV-1 sequences including 34 sequences from our study site, 30 sequences from the surrounding state of Antioquia, 4 sequences representing genotype I, 1 sequence representing genotype II, 5 sequences representing genotype IV and 12 sequences representing genotype V were used as input for Bayesian analysis.

A Maximum Clade Credibility tree was reconstructed using a Markov Chain Monte Carlo (MCMC) approach as implemented by the program BEAST v1.8, under a HKY + Γ + Ι model and otherwise default parameters. The MCMC chains were run for at least 10 million generations, sampled every 1000 steps. A maximum clade credibility tree was generated using Tree Annotator (BEAST package) with the initial 10% of steps removed as burn-in [33].

### Statistical analysis

All data were analyzed with GraphPad Prism software version 6.00 for Mac OS X (GraphPad Software, Inc.). A two-way ANOVA followed by Tukey’s multiple comparison test with confidence levels at 0.05 was used to test the null hypothesis that the mean ranks of synonymous and nonsynonymous SNPs (frequency ≥1%) between genes were the same among all pooled samples. A One-way ANOVA (Kruskal-Wallis test followed by Dunn’s multiple comparison test with confidence levels at 0.05) was used to test the null hypothesis that the mean ranks of synonymous and nonsynonymous SNPs between the two clades discovered are the same. A multiple t-test using the Holm-Sidak method to correct for multiple comparisons with alpha = 5% to determine statistical significance was used to test the differences in nucleotide diversity (π) per gene between clades. An unpaired t-test (Mann-Whitney test P<0.05) was used to compare mean πN/πS ratios per gene between clades. Finally, we assessed differences in pooled average πN and πS values within each clade using a paired t-test (Wilcoxon signed-rank test P<0.05).

## Results

**Fig 1:**
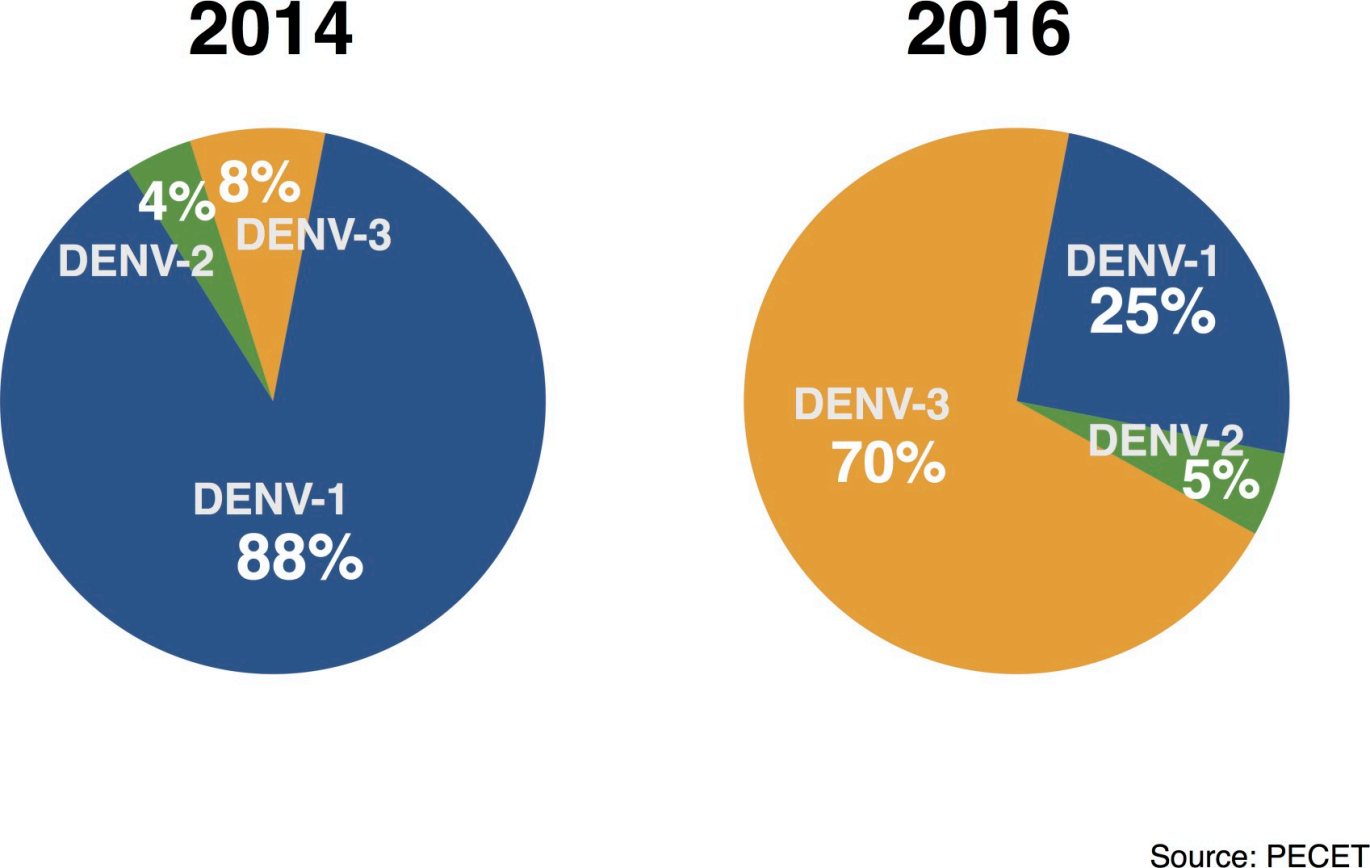
Barrio Paris, Colombia. **DENV serotype distribution.** The proportion of laboratory confirmed dengue cases by serotype from the fever clinic in the Paris neighborhood of Bello, Colombia. Surveillance began May of 2014 and ended in January of 2016.

**Fig 2:**
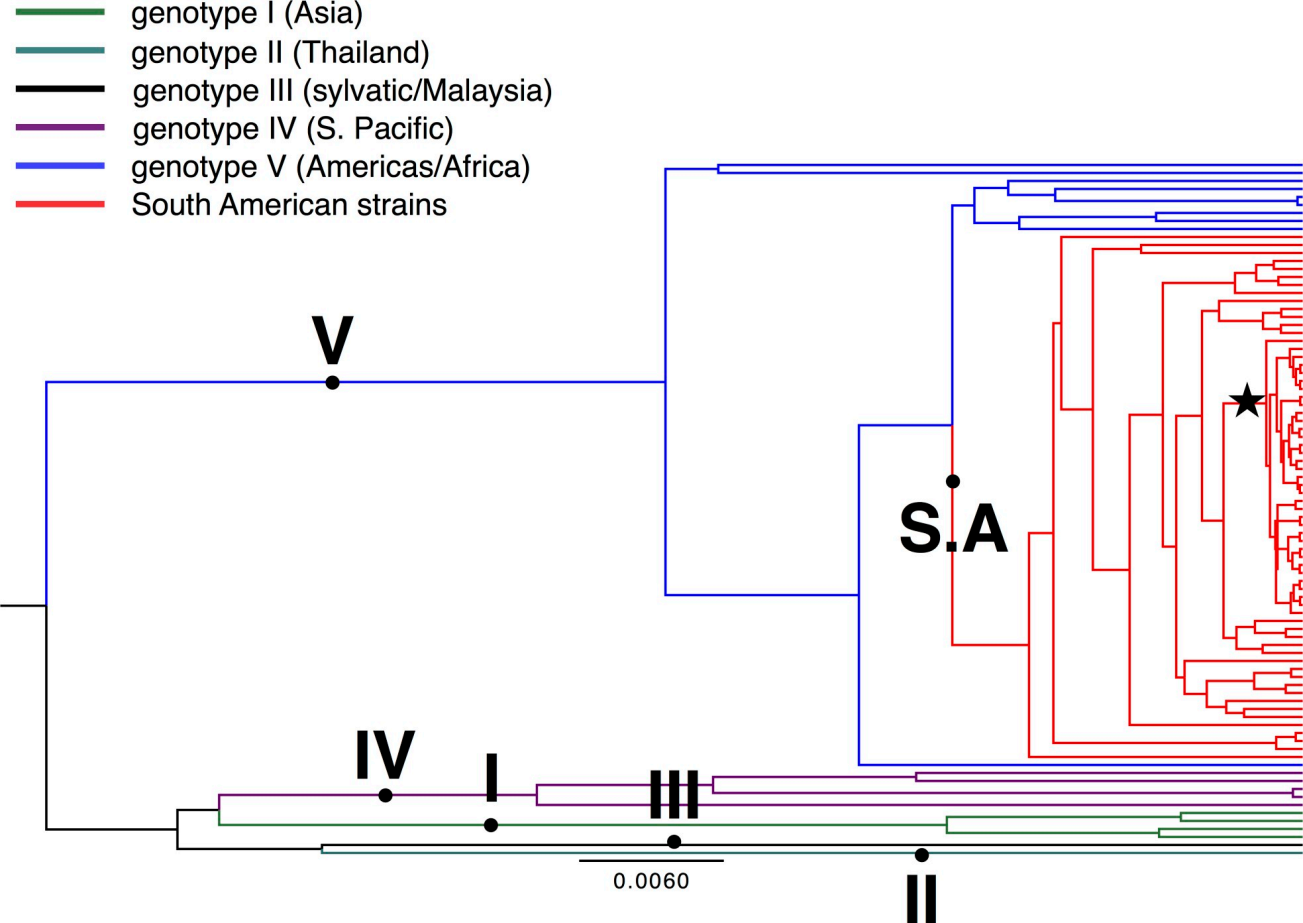
Genotyping of Colombian DENV-1 sequences based on E gene. Bayesian Maximum Clade Credibility tree of 87 DENV-1 E gene nucleotide sequences was reconstructed using a Markov Chain Monte Carlo (MCMC) approach as implemented by the program BEAST v1.8, under a HKY + Γ + Ι model and otherwise default parameters. Sequences representative of all five DENV-1 genotypes included. Only nodes (circles) with at least 60% posterior probability support are shown. Tree branches were transformed to cladogram for ease of visualization. Nodes representing the five DENV-1 genotypes are highlighted. Genotypes I-V are displayed by different color branches detailed in the legend. S.A stands for South American strains. The clade containing all strains sequenced in this study is marked by a star.

To analyze DENV-1 between/within-host (inter/intra) viral diversity present in humans, we deep-sequenced 34 DENV-1 viruses from sera of infected patients from the Paris neighborhood of Bello, Colombia (Table 1). Our study period was characterized by an abundance of DENV-1 infections throughout 2014, an inter-epidemic period in 2015, and finally a decline in DENV-1 prevalence during a rise in DENV-3 prevalence throughout 2016 (Fig 1). We assembled E gene consensus sequences from each patient to determine the degree of genetic variability among viruses by Bayesian phylogenetic approaches. These sequences were aligned with 46 other DENV-1 E sequences available on NCBI GenBank representing all major DENV-1 global genotypes, frequently used for phylogenetic analyses. Our reconstructed phylogeny (Fig 2) clustered all 34 DENV strains within Genotype V (America/Africa), alongside other South American strains. Results from our analyses are in accordance with genotyping data reported from South America in previous years [21,26,34–36].

### Evidence for strain turnover within DENV-1

**Fig 3:**
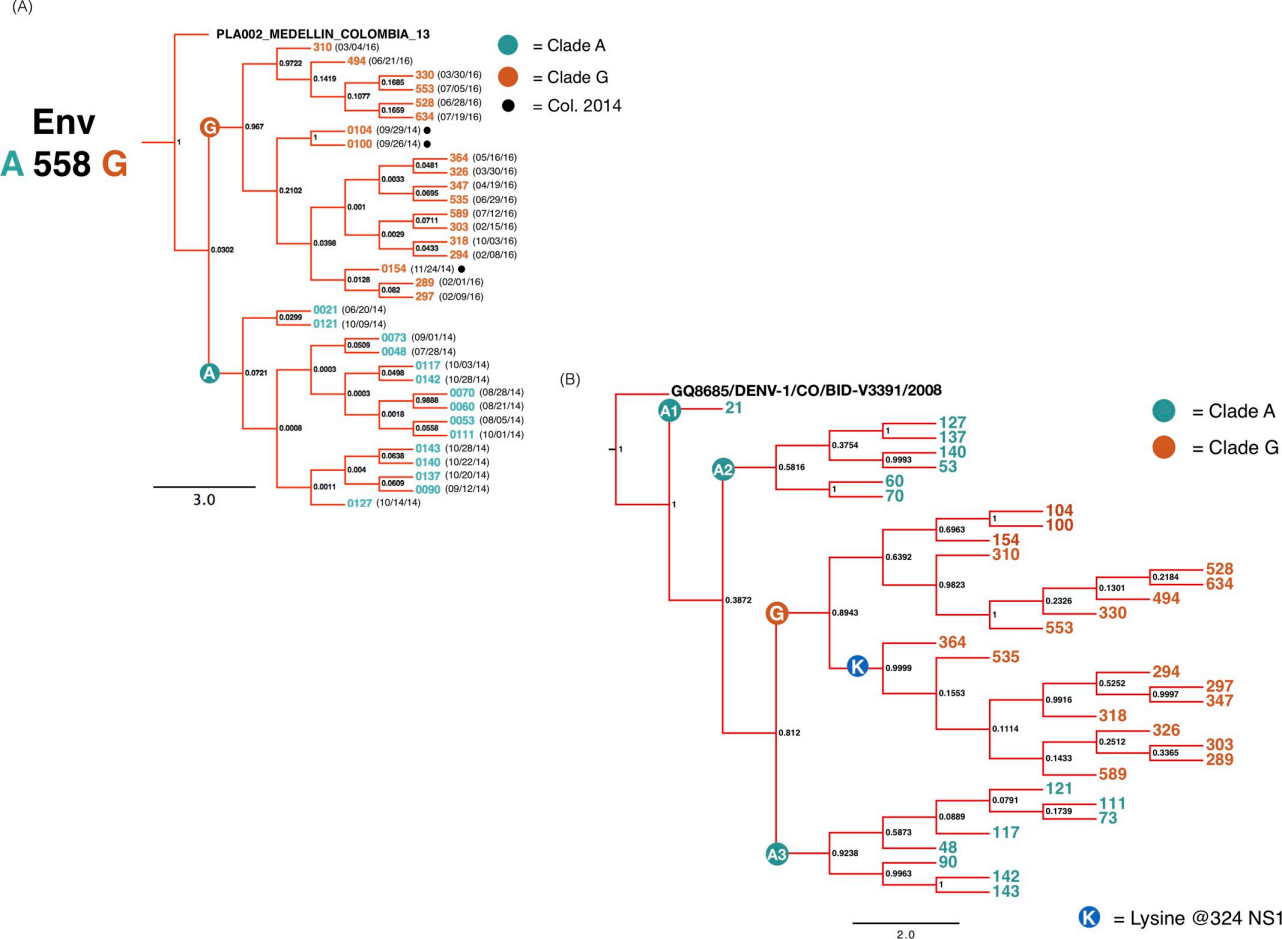
Phylogenetic relation of DENV-1 sequences collected at the Paris neighborhood fever clinic. (A) A closer look at the clade containing all DENV-1 sequenced from our study site (marked by star) in Fig 2. Bayesian Maximum Clade Credibility tree of 34 DENV-1 E gene nucleotide sequences from viruses collected at our study site reconstructed using a Markov Chain Monte Carlo (MCMC) approach as implemented by the program BEAST v1.8, under a HKY + Γ + Ι model and otherwise default parameters. Dates formatted in (mm/dd/yy). (B) Whole genome based phylogenetic relation. Bayesian Maximum Clade Credibility tree of 34 DENV-1 complete protein coding sequences from viruses collected at our study site reconstructed using a Markov Chain Monte Carlo (MCMC) approach as implemented by the program BEAST v1.8, under a HKY + Γ + Ι model and otherwise default parameters.

Upon further analysis, our E-based phylogeny segregated all sequenced viruses into two distinct clades (Fig 3A). Sequence comparison identified that a synonymous A to G substitution at position 558 (E:A558G) was responsible for the separation of the clades. For ease of comparison, these two clades have been designated “clade A” and “clade G.” Interestingly, all viruses collected in 2014 belonged to clade A, except for three samples (100, 104, 154) collected in the last third of the year (Table 1). These three viruses harbor a guanine at position 558, suggesting that this synonymous variant, later fixed among viruses collected at our site in 2016, may have begun circulating as early as September of 2014.

Due to low innate variability within E [2,4,17,20] and the short time period between sample collections, much of the derived branching pattern was not highly supported. In order to achieve a more robust phylogeny and explore variation between viruses at a whole-genome level, we performed the same phylogenetic analysis using the entire genome coding region (Fig 3B). The most recent DENV-1 complete genome sequence from Colombia (2008; GenBank #GQ868570) served as the outgroup as it diverges enough to provide high statistical support. This improved the statistical power across several nodes, but not all, likely due to low overall diversity among circulating viruses in this study. Clade G remained unified (PP = 0.812) while clade A viruses split into three separate clades (A1 = 1, A2 = 0.387, A3 = 0.812). One synonymous nucleotide substitution, T1701C within NS5, differentiated clade A3 from the rest.

### Lysine at position 324 of NS1 distinguishes a subclade within clade G

**Fig 4:**
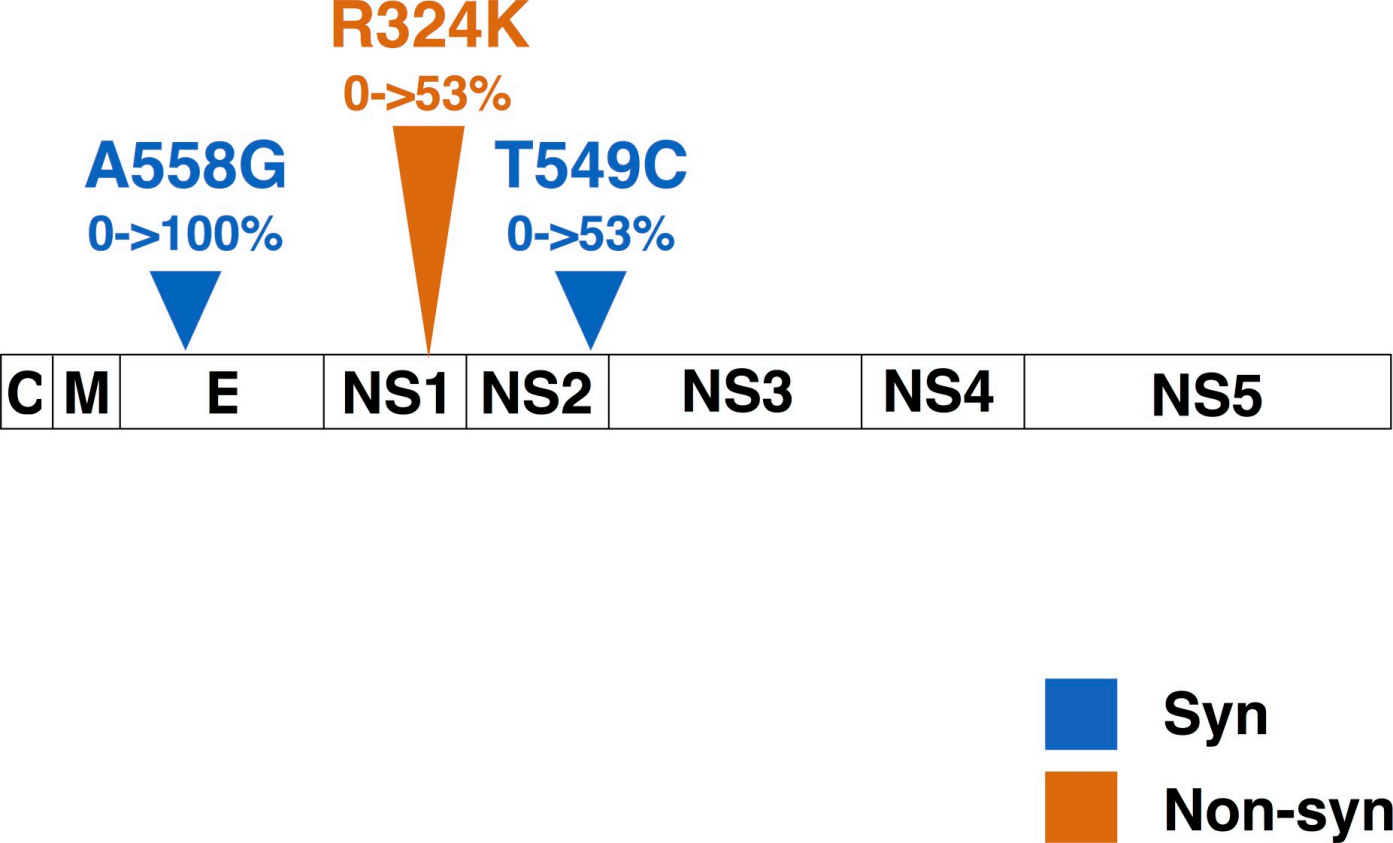
High frequency de-novo variants across the CDS associated with clade A to G replacement. Consensus CDS comparison of Clade A vs. G samples. Nonsynonymous mutation R324K in NS1 and synonymous mutation T549C in NS2A undetected in Clade A, observed in 10/9 samples composing Clade G.

Comparison of complete consensus sequences between clade A and clade G shows that E:A558G was the only nucleotide change that became fixed along with the clade replacement. A non-synonymous mutation, R324K in NS1 (NS1:R324K), and one synonymous mutation, T549C in NS2A (NS2A:T549C), were both absent in clade A samples and present in 53% (10/19) of clade G samples (Fig 4), suggesting that these variants could be increasing in frequency throughout the population. We compared consensus sequences within each clade to assess the overall variance. Notably, only a few nucleotide variants were shared at the consensus level by more than 2 samples in each clade. Clade A had one variant shared by 7/15 samples while clade G had 5 variants shared by more than 25% of samples. Interestingly, a synonymous variant observed in two clade G samples at the consensus level was also detected at low frequency (1.9%) within one sample (142) from clade A (Table 2). No other variants present in clade G were detected as low-frequency variants among clade A samples (S1 Table).

**Table 2:** Consensus polyprotein region comparison among each clade.

**Clade A (n = 15)**					
**Position (CDS)**	**Clade** **Consensus**	**Variant**	**Frequency %**	**Amino acid change**	**Gene**
462	C	T	13		prM
1131	G	A	13		Envelope
2962	G	T	13		NS1
3365	G	A	20	R to K	NS2A
7230	A	G	13		NS4B
8646	A	G	13		NS5
9180	C	T	47		NS5
**Clade G (n = 19)**					
**Position (CDS)**	**Clade** Consensus	**Variant**	**Frequency %**	**Amino acid change**	**Gene**
411	C	T	26		prM
860	G	C	11		Envelope
1164	C	T	32		Envelope
3296	A	G	47	K to R	NS1
3930	C	T	47		NS2A
4697	C	T	11	T to M	NS3
*6535	T	C	11		NS4A
7005	G	A	21		NS4B
7605	A	G	26		NS5

* Observed as low frequency variant in sample 142 of clade A

Viruses harboring a lysine at position 324 of NS1 form a sub-clade within clade G (Fig 3B). The NS1 gene encodes an essential, multifunctional protein that is secreted extracellularly and plays a role in immune evasion [37,38]. DENV genetic studies have largely focused on the E gene, with less known about NS1 genetic plasticity. Amino acid 324 of NS1 is located along the distal tip of the -ladder domain, a region that is exposed in its soluble-secreted hexameric form. This site is not conserved among flaviviruses [39,40], and could potentially interact with host immune factors[39]. NS1 sequence comparison among flaviviruses performed by other groups suggests that the arginine present in clade A viruses is similar to globally circulating DENV-2 strains while the lysine present in clade G viruses resembles other globally circulating DENV-1 viruses [39,40]. Viruses belonging to clade G have a diverse spatial distribution within our study site and are found throughout the sampling period which suggests that the lysine-containing sub-clade is not an artifact of sampling bias.

### Nonsynonymous SNPs/site in NS3 higher among clade G samples

**Fig 5:**
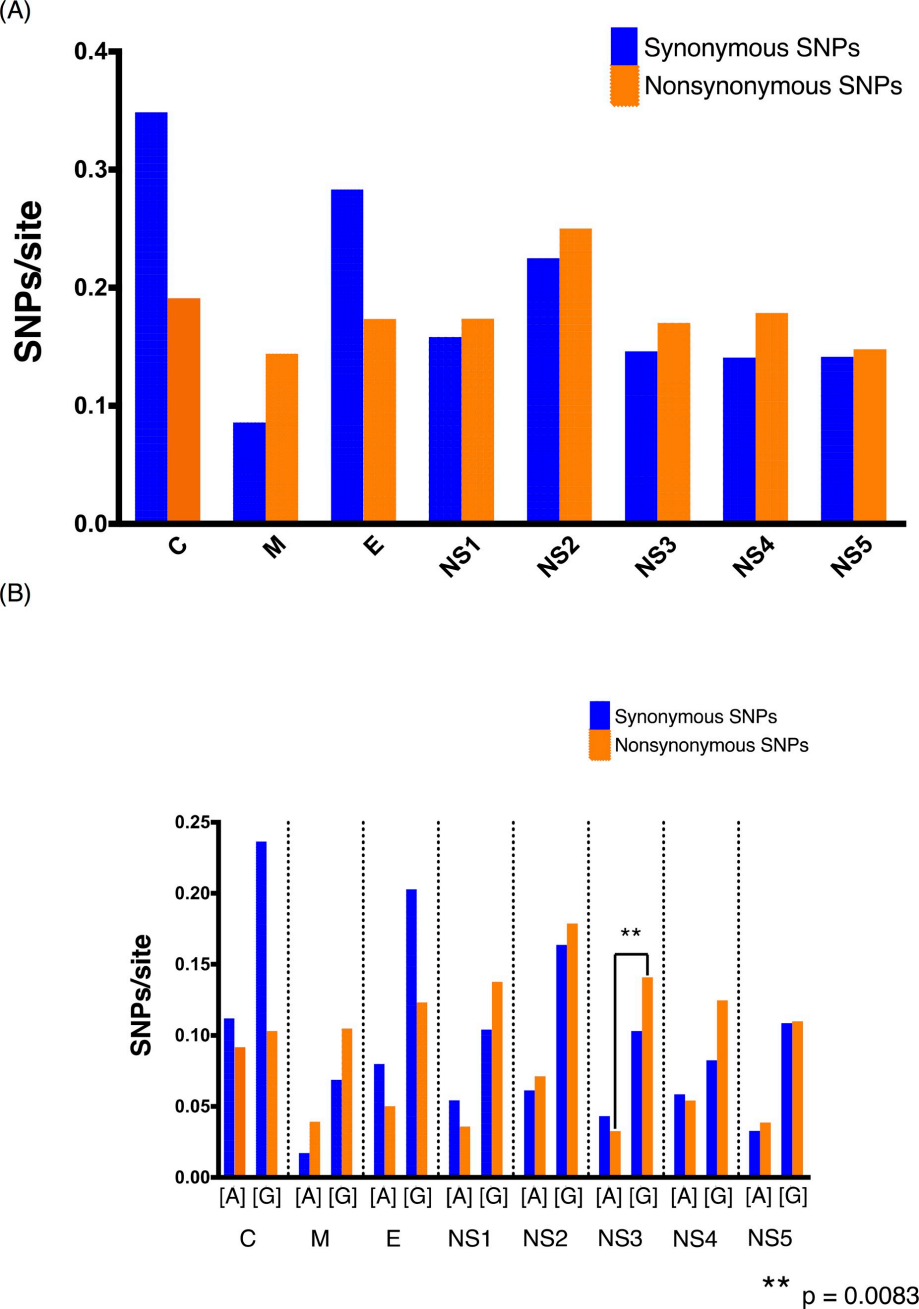
Significant increase in NS3 nonsynonymous SNPs/site associated with strain turnover. The repertoire of SNPs present above 1% within-hosts was visualized as a pooling of all samples (A) as well as a side by side comparison of clade A vs. G (B). SNP count was normalized to the number of synonymous and nonsynonymous sites per gene. Genes are separated by a dotted line across the plot and identified by initials below the axis. There were no significant differences between genes when all samples were pooled. A significant increase in nonsynonymous SNPs/site is observed across NS3.

Deep sequencing identified a variety of intra-host minority variants (present at ≥ 1.0% frequency and < 50%) in infected individuals scattered throughout the viral genome; yet the majority were unique to individual patients, suggesting that they likely did not mediate significant adaptation at the population level. A detailed presentation of variants detected across all samples are reported in Supplementary Tables 1 and 2. Next, we analyzed the total number of synonymous and nonsynonymous SNPs present per gene, independent of frequency. We plotted a pooling of all samples as well as a side by side comparison of E designated clade A vs. G to explore possible differences among strains. The raw frequencies of synonymous and nonsynonymous substitutions per site showed no significant differences between genes when all samples were pooled (Fig 5A, two-way ANOVA followed by Tukey’s multiple comparison test). However, a comparison of clade A vs. G revealed a pattern of elevated nonsynonymous SNPs/site among non-structural (NS) genes in clade G, though this pattern was only significant for NS3 (Fig 5B, P = 0.0083, one-way ANOVA followed by Dunn’s multiple comparison test).

### Significant changes in diversity indicate positive selection acting within NS1

To explore selection pressures acting within different genes as well as between clades, we first evaluated the overall genetic variability, taking into account SNP frequency, across the entire DENV-1 genome. Nucleotide diversity was calculated as the average number of pairwise synonymous and nonsynonymous differences (πS and πN, respectively) per site across all 3 structural and 5 nonstructural genes. To account for differences in coverage among samples, as well as uneven coverage across the genome within samples, π was estimated using assemblies that were subsampled to a set coverage of 500x per base (see Methods). πN/πS ratios significantly > 1 are indicative of diversifying selection favoring new polymorphisms. πN/πS ratios < 1 are suggest that purifying selection is acting to remove new variation. πN/πS ≈ 1 indicates neutral evolution, or that genetic drift is playing the predominant role in shaping variation at that locus.

**Fig 6:**
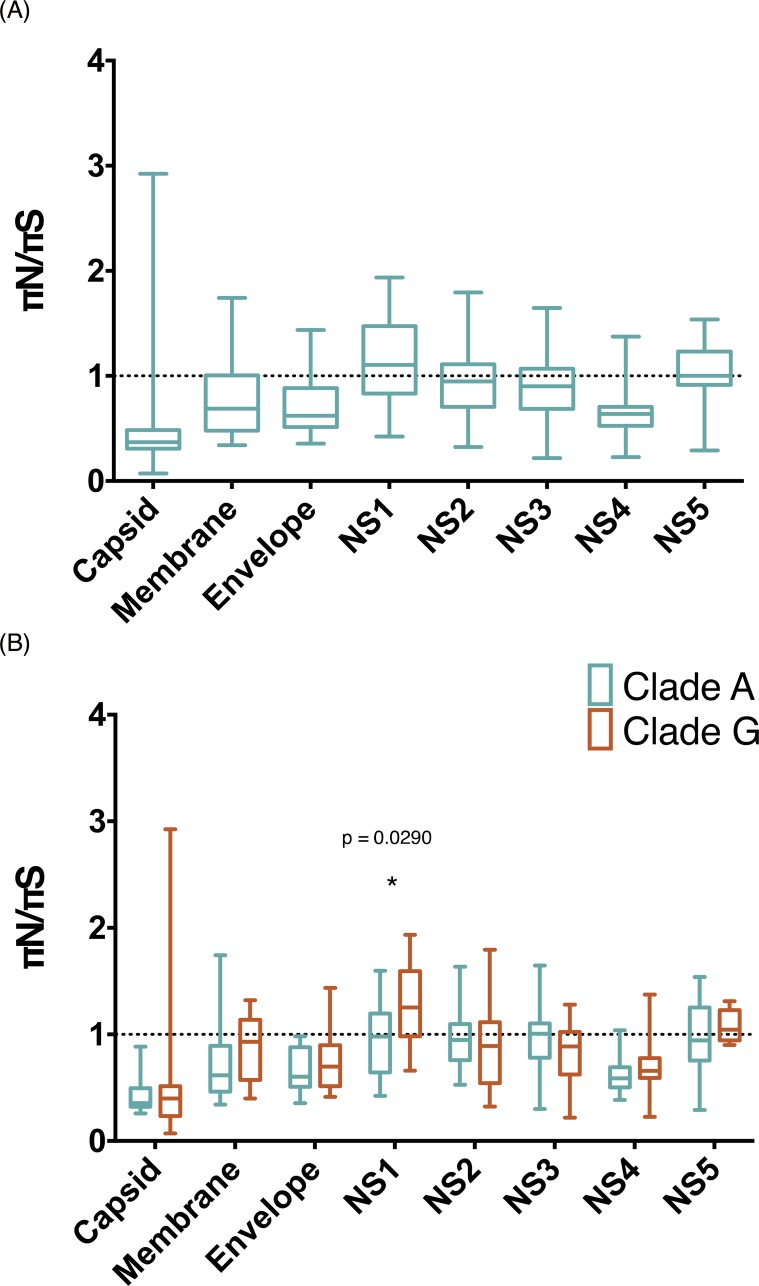
Statistically significant increase in average πN/πS ratio between clades A and G. The πN and πS statistic were calculated for each gene using assemblies subsampled to 500x coverage across the CDS for each sample. The average value for each gene across each clade is displayed as a box and whisker (min to max) plot. (A) All samples pooled into one data set. (B) Comparison between samples containing E:558Avs.G. We found a statistically significant difference in the average πN/πS ratio of the NS1 gene (P = 0.029; Student’s t-test) between groups A and G (πN/πS ratio = 0.960 and, 1.284 for clades A and G, respectively).

First, we calculated average πN/πS ratios for each gene of each separate sample. We pooled all of our samples as well as binned our results into samples containing E:558A and E:558G. Pooling all samples showed a trend toward higher πN/πS ratios in non-structural genes vs. structural genes, consistent with the idea that structural genes may be under stronger purifying selection than nonstructural genes. We observed neutral evolution within NS5 and values marginally suggesting positive selection within NS1 (Fig 6A). Comparing samples that harbored an A vs. G at position E:558 revealed a statistically significant difference in the average πN/πS ratio of NS1 (P = 0.029; Student’s t-test) (πN/πS ratio = 0.960 and 1.284 for groups A and G, respectively) (Fig 6B). Both groups exhibit similar πN values within the NS1 gene (mean πN = 0.0005623 and 0.0005721 for A and G, respectively), but group G had a lower average πS (mean πS = 0.0006331 and 0.0004436 for group A and G, respectively) (Table 3).

**Table 3:** Comparison of mean nonsynonymous to synonymous nucleotide diversity by gene between clade A and G samples.

**Clade A**				
**Gene**	**πN/πS**	**Mean πN ± SD**	**Mean πS ± SD**	**Significance**
Capsid	0.4139939	0.0003275 ± 7.586e-005	0.0008685 ± 0.0003333	***
Membrane	0.7129871	0.0003835 ± 0.0001523	0.000585 ± 0.0002230	**
Envelope	0.6784652	0.0005142 ± 0.0001248	0.0008075 ± 0.0002817	***
NS1	0.9598823	0.0005623 ± 0.0001841	0.0006331 ± 0.0002103	ns
NS2	0.9553852	0.0005355 ± 0.0001814	0.000585 ± 0.0002072	ns
NS3	0.9364279	0.0004595 ± 8.634e-005	0.0005898 ± 0.0003242	ns
NS4	0.6240892	0.0004514 ± 0.0001093	0.0007775 ± 0.0002946	***
NS5	0.9688271	0.0004919 ± 0.0001411	0.0005903 ± 0.0003747	ns
**Clade G**				
**Gene**	**πN/πS**	**Mean πN ± SD**	**Mean πS ± SD**	**Significance**
Capsid	0.5676073	0.0003658 ± 0.0003948	0.0007219 ± 0.0005372	*
Membrane	0.866891	0.0003604 ± 0.0001469	0.0004241 ± 0.0002079	ns
Envelope	0.7446317	0.0005693 ± 0.0002042	0.0008282 ± 0.0003266	**
NS1	**1.28427**	0.0005741 ± 0.0002760	0.0004436 ± 0.0001526	*
NS2	0.9103566	0.0004609 ± 0.0001544	0.000576 ± 0.0003398	ns
NS3	0.8470698	0.0004229 ± 0.0001817	0.0006592 ± 0.0005134	*
NS4	0.7082008	0.0004788 ± 0.0002515	0.0007832 ± 0.0005533	***
NS5	**1.077734**	0.000507 ± 0.0001821	0.000472 ± 0.0001410	ns

significant

* < 0.05

** < 0.01

*** < 0.001

**πN/πS > 1: new polymorphism is favored**

πN/πS < 1: purifying selection (new polymorphism is removed)

πN/πS = 1: neutral evolution

We looked further into πN/πS ratios per gene between groups A and G in search of differences in selection pressure associated with strain turnover. We expected to see higher πN values among non-structural genes which are less exposed to immune pressures while the fixed polymorphism observed in E, historically exposed to immune pressures, was expected to be associated with selection. The NS1 and NS5 genes had the highest πN/πS ratios overall, NS1 being slightly higher (Table 3). The NS5 πN/πS ratio was higher in group G (πN/πS = 1.078) than in group A (πN/πS = 0.969), but not significantly so. The capsid (C), membrane (M) and non-structural gene 4 (NS4) had the lowest diversity values throughout both clades. All other genes also showed no significant difference in diversity between clades and had values indicative of purifying selection (Fig 6B and Table 3). Surprisingly, we did not detect significant changes in πN/πS values within E, which is the major surface glycoprotein. Epitopes along E have been reported to be the major targets of humoral responses against DENV infection [41–44]. However, our resulting πN/πS values are not suggestive of changes in selection pressure associated with strain turnover nor are they indicative of polymorphisms being favored within this gene in either group.

Mean πN and πS values were also calculated per gene in order to display differences between clades regardless of high or low πN/πS ratios. Mean πS values across the E gene are significantly larger (P <0.001 and <0.01 for group A and G respectively) than mean πN values in both clades, but not considerably larger than any other gene. Our assessments of nucleotide diversity are consistent with what has been reported in the literature previously, i.e., purifying selection acts across most of the DENV genome [2,4,14,17–19,45,46].

### Strain turnover associated with elevated diversity at a subset of sites

**Fig 7:**
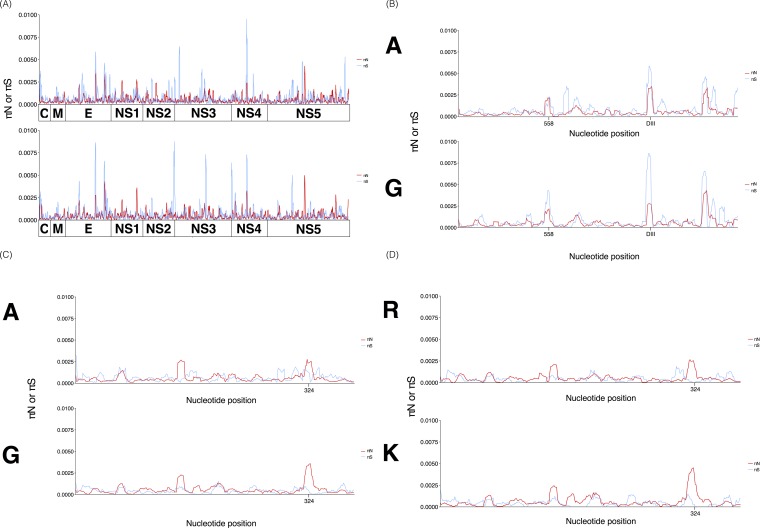
Sliding window analysis reveals peaks in DENV-1 synonymous and nonsynonymous diversity. To ensure that strong signals of positive selection were not diluted out by averaging values across entire genes, we examined πN and πS values using a sliding window approach. (A) We performed a pooled sliding window analysis grouping samples into clades in order to identify any apparent peaks along the genome. Clade A is displayed on the top panel, Clade G at the bottom. The strongest peaks of synonymous variation were observed in E, NS3 and NS4, while strong peaks of nonsynonymous variation were observed in NS1 and NS5. πN is shown as a red line, πS is shown as a blue line. A depiction of the DENV gene organization lies below the x axis. (B) An enlarged rendering of the graph in (a) spanning the E gene. Nucleotide position 558 where a synonymous SNP became fixed is highlighted. The major peak in diversity within both clades is highlighted as part of domain III (DIII). (C) An enlarged rendering of the graph in (a) spanning the NS1 gene. Amino acid position 324 is highlighted. (D) An enlarged rendering of clade G from the graph in (a) spanning the NS1 gene, divided into samples containing an arginine (R) or lysine (K) at amino position 324.

To further explore differences in non-synonymous and synonymous variation across clades and ensure that strong signals of positive selection were not diluted out by averaging values across entire genes, we examined πN and πS values using a sliding window approach. First, a pooled sliding window analysis was performed grouping samples into clades in order to identify any apparent peaks in diversity along the genome. As expected, most nucleotide positions along the DENV genome displayed low levels of diversity. However, a number of synonymous and nonsynonymous peaks across the genome were apparent. Peaks of synonymous variation governed the landscape of E, NS3 and NS4, while strong peaks of nonsynonymous variation were observed in NS1 and NS5 (Fig 7A).

The E gene was governed by synonymous diversity (Fig 7B) and displayed two interesting peaks. A peak around position 558, a non-exposed site located at the center of the protein within domain I, and a large signal of synonymous diversity coming from within domain III, the most exposed portion of the glycoprotein and major target of cell mediated immunity [41,47]. In contrast, large peaks along NS1 were dominated by nonsynonymous diversity (Fig 7C). Interestingly, nearly half the samples in clade G displayed a nonsynonymous variant (R or K) at amino acid position 324 (nucleotide position 971). We used the sliding window approach to gauge individual contributions made by each variant group. Samples containing an arginine (R) at this site displayed similar levels of nonsynonymous diversity to clade A (πN~0.0025), while samples containing a lysine (K) at this site displayed almost twice that (πN~0.0050) (Fig 7D). On average, NS1 exhibits higher levels of nonsynonymous diversity in comparison to the rest of the genome.

## Discussion

In general, DENV transmission cycles in South America are characterized by years of alternating dominance of a single serotype that fluctuate every 3–5 years [21,22]. The interplay between shifts in serotype dominance patterns and evolutionary drivers of serotype extinction as well as replacement of whole lineages within DENV serotypes has not been explored. Here, full genome, deep sequencing was used to characterize DENV-1 clade replacement coincident with a shift in serotype dominance from DENV-1 to DENV-3. Surprisingly, our analysis of within-host viral genetic diversity found evidence for diversification in the NS1 gene. In contrast to the hypothesis that the gene encoding the major glycoprotein would be the primary target of diversifying selection to avoid immune targeting, E genes in our dataset displayed levels of nonsynonymous to synonymous diversity indicative of purifying selection during seasons of both DENV-1 dominance and decline. Furthermore, the strain turnover observed in this study was associated with a significant increase in the ratio of nonsynonymous to synonymous variation within NS1. Interestingly, this increase in πN to πS ratio is largely due to a reduction in synonymous mutations in the latter clade. While one would expect diversifying selection to be associated with an increase in nonsynonymous mutations, this data opens questions regarding the attenuation of synonymous diversity, its possible mechanisms and interpretation.

Signals of diversifying selection are compatible with the biological role of NS1 in DENV replication and transmission. NS1 is found both as a dimer within the infected cell, where it is involved in replication, and as a secreted hexameric complex involved in immune evasion [37,39]. This multifaceted protein is also involved in pathogenesis, assembly/release of infectious particles and replication[48]. The protective immune response against DENV is believed to be primarily humoral, with the most potent neutralizing antibodies targeting E, the membrane-associated protein (prM), and NS1 [43]. In fact, antibodies against NS1 alone have shown protection against subsequent DENV infection [42,49,50]. We found putative evidence for diversifying positive selection within NS1, which is consistent with an expanding DENV-1 specific immune response after pronlonged serodominance in the area and could explain an increase in nonsynonymous pressure at an exposed site like NS1:R324K over time. The progression of specificity and cross-reactivity regarding sequential heterologous DENV humoral responses remains a mystery. Antibodies produced following DENV infection have an initial period of cross-serotype neutralization lasting several months [51]. Although homologous DENV protection is thought to be long-lasting following primary infection, there is some evidence of homotypic reinfection, which challenge the current understanding of DENV immunity[10]. We do not fully understand how secondary infections shape the DENV antibody landscape. Regardless, immune pressures could result in selection for NS1 amino acid diversity during vertebrate host replication, though this requires further experimental confirmation/verification.

The serotype shift observed within the Paris neighborhood is supported by an existing pattern of single serotype dominance that alternates every 3–5 years. This serotype shift lagged behind the epidemiological pattern detected across the state as a whole. Antioquia’s 2008–2012 dengue season was undoubtedly dominated by DENV-1 and was both preceded and followed by seasons of DENV-3 dominance (Rita Almanza, personal communication). In 2014, the state of Antioquia’s surveillance efforts detected DENV-3 as the dominant serotype (Rita Almanza, personal communication); whereas, the Paris neighborhood reported mostly DENV-1 infections. It was not until after June of 2016 that DENV-3 prevalence increased in patients sampled in the Paris neighborhood fever clinic. This lag could be explained by localized density-dependent transmission of DENV. For example, DENV-1 tends to move from urban to less densely populated areas, while densely populated regions act as major transmission foci [7]. The vast majority of individuals that reside within the Paris neighborhood and Bello as a whole generally migrate towards metropolitan areas for work. The DENV incidence patterns observed in the nearby city of Medellin are often directly in phase with reports for the whole state of Antioquia. It is likely that inhabitants from the Paris neighborhood who migrate to Medellin for work, spending most of their day at this location, become infected with the dominant serotype of the metropolitan area and slowly introduce novel viruses into their home community [7,52,53]. This transmission model could likely ressemble similar commuting populations across other departments as well as neighboring countries. The fact that DENV infection patterns observed in the Paris neighborhood lagged behind seroprevalence reported in Medellin, and the state of Antioquia, suggests the possibility of introduction from the metropolitan area to our study site; perhaps only noticeable after the majority of the Paris population is no longer susceptible to locally circulating strains.

Historically, the majority of DENV sequencing efforts near our study site, and in Latin America in general, have focused largely on E [16]. Arboviruses such as DENV have inherently low variation rates within this gene [14,17], which often result in weak phylogenetic reconstructions when using samples from a short temporal period. Hence, our initial phylogenetic reconstruction based off E was not robust, but clearly defined two separate clades (Fig 3A). This assessment was satisfactory for genotyping our viruses, but not sufficient for confidently characterizing strain turnover during our sample collection timeline. A robust reconstruction was only achieved when using the complete viral coding sequence (Fig 3B). However, a whole genome approach was not feasible for genotyping samples, because whole genome DENV sequences from all genotypes as well as surrounding areas are scarce while the majority of Latin American sequences publicly available were uploaded thanks to an initiative from the BROAD institute [54]. This two-part phylogenetic analysis sheds light on the genetic diversity that can be missed when only utilizing a low-variability gene like E, as well as the necessity for further initiatives to implement whole genome NGS in Latin America.

Whole genome phylogenetic reconstruction confirmed the occurrence of strain turnover within our study site and identified interesting patterns of sequence variation within the observed clades. In a hyperendemic area such as our study site, one reasonable hypothesis explaining strain turnover could be that selection is acting to produce changes capable of combating rising herd immunity. Another plausible scenario is that independent of human immunity, strain replacements favor viruses best equipped for non-human seasonal changes such as weather, mosquito populations and competing pathogens. We did not find evidence of selection acting within E, historically the most likely candidate of immune pressure. Also, the fixed nonsynonymous polymorphism we observed outside this gene had only marginal evidence of positive selection. In this area, it is likely that strain replacement did not occur due to classical immune evasion.

Our study corroborates multiple recent findings while providing insight into DENV evolutionary mechanisms not previously observed through frequent large-scale analyses. DENV-1 strain extinction and replacement were observed during periods of co-circulation and shifting prevalence among serotypes [6,17], and specifically associated with a decline in DENV-1 prevalence with strain turnover which is consistent with previous findings [19]. In addition, we observed similar levels of within-host nucleotide diversity across the DENV genome while more low frequency variants were found in NS genes. Both of these observations have been made by groups utilizing DENV whole genome analyses [4,45]. More interestingly, our findings are in agreement with other flaviviruses. For example, Erhbar et al. found similar signals of diversity and selection across E and NS1 of West Nile viruses (WNV) sequenced from mosquito samples [55], as well as an overlapping, highly polymorphic region among our studies, comprised primarily of non-synonymous variants within NS1. Although it is not known whether selective pressures for DENV differ between human and mosquito hosts [5,55,56], our data suggest a common region capable of tolerating high levels of genetic diversity. However, further study into the origin of mosquito- versus human-specific selective pressures is needed to map areas of genetic plasticity with certainty.

In summary, our results suggest that whole genome analysis is necessary when attempting to understand factors shaping DENV evolution during periods of simultaneous serotype and clade replacement. Additionally, our analyses emphasize the importance of the scale at which an affected population is assessed. Our local, intra-epidemic comparison of whole genome deep sequencing data revealed patterns that may be missed by common broad geo-temporal analyses. These results highlight patterns of genetic diversity indicative of selection among nonstructural genes thereby placing importance on sequencing efforts outside the E gene, specifically among NS1. Continued intra-epidemic surveillance of dengue using modern methods is salient to understanding the viral contributions to naturally occurring cycles of serotype and clade replacement. Such information is of utmost importance towards both understanding the constraints of DENV evolution in humans as well as assessing the effects of control programs at the population level.

## Supporting information

S1 Fig RAlmanza PC. Rita Almanza, personal communication. Percentage of Dengue serotypes circulating in the state of Antioquia, Colombia 1992–2014.
(TIFF)

S1 Table Clade A variants. Variants above 1% frequency present in Clade A samples.
(XLSX)

S2 Table Clade G variants. Variants above 1% frequency present in Clade G samples.
(XLSX)
