# Supplementary figures and images for: Tracking dengue virus type 1 genetic diversity during lineage replacement in an hyperendemic area in Colombia
Source: PLoS One. 2019 Mar 7;14(3):e0212947. doi: 10.1371/journal.pone.0212947 (PMC6405123; doi:10.1371/journal.pone.0212947)

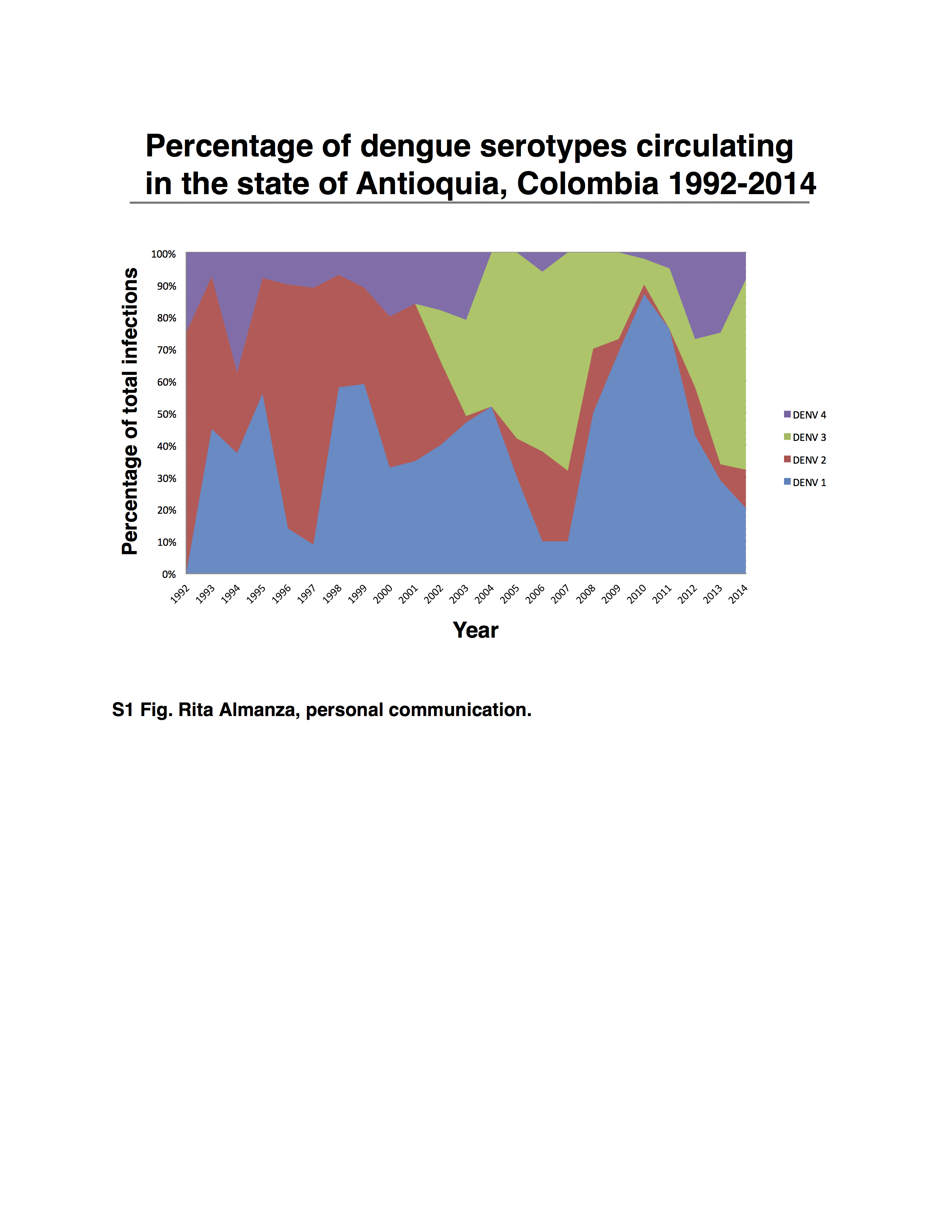

Supplement: S1 Fig — Rita Almanza, personal communication. Percentage of Dengue serotypes circulating in the state of Antioquia, Colombia 1992–2014. (TIFF) [file pone.0212947.s001.tiff]
